# Association of prior outpatient diabetes screening with cardiovascular events and mortality among people with incident diabetes: a population-based cohort study

**DOI:** 10.1186/s12933-023-01952-y

**Published:** 2023-08-28

**Authors:** Calvin Ke, Anna Chu, Baiju R. Shah, Sheldon Tobe, Karen Tu, Jiming Fang, Haris Vaid, Peter Liu, Aishah Cader, Douglas S. Lee

**Affiliations:** 1 Department of Medicine, University of Toronto, Toronto, ON Canada; 2 Department of Medicine, Toronto General Hospital, University Health Network, Toronto, ON Canada; 3 ICES, Toronto, ON Canada; 4 Department of Medicine, Sunnybrook Hospital, Toronto, ON Canada; 5 Northern Ontario School of Medicine, Sudbury, ON Canada; 6 Department of Family and Community Medicine, University of Toronto, Toronto, ON Canada; 7 North York General Hospital and Toronto Western Family Health Team, University Health Network, Toronto, ON Canada; 8 Division of Cardiology, University of Ottawa Heart Institute, Ottawa, ON Canada; 9 Department of Public Health Sciences School of Medicine, Queen’s University, Kingston, ON Canada; 10 Peter Munk Cardiac Centre and Ted Rogers Centre for Heart Research, Toronto, ON Canada

**Keywords:** Diabetes screening, Targeted screening, Cardiovascular disease, Mortality, Age at diagnosis, Population-based study

## Abstract

**Background:**

Outcomes of diabetes screening in contemporary, multi-ethnic populations are unknown. We examined the association of prior outpatient diabetes screening with the risks of cardiovascular events and mortality in Ontario, Canada.

**Methods:**

We conducted a population-based cohort study using administrative databases among adults aged ≥ 20 years with incident diabetes diagnosed during 2014–2016. The exposure was outpatient diabetes screening performed within 3 years prior to diabetes diagnosis. The co-primary outcomes were (1) a composite of all-cause mortality and hospitalization for myocardial infarction, stroke, coronary revascularization, and (2) all-cause mortality (followed up until 2018). We calculated standardized rates of each outcome and conducted cause-specific hazard modelling to determine the adjusted hazard ratio (HR) of the outcomes, adjusting for prespecified confounders and accounting for the competing risk of death.

**Results:**

We included 178,753 Ontarians with incident diabetes (70.2% previously screened). Individuals receiving prior screening were older (58.3 versus 53.4 years) and more likely to be women (49.6% versus 40.0%) than previously unscreened individuals. Individuals receiving prior screening had relatively lower standardized event rates than those without prior screening across all outcomes (composite: 12.8 versus 18.1, mortality: 8.2 versus 11.1 per 1000 patient-years). After multivariable adjustment, prior screening was associated with 34% and 32% lower risks of the composite (HR 0.66, 0.63–0.69) and mortality (0.68, 0.64–0.72) outcomes. Among those receiving prior screening, a result in the prediabetes range was associated with lower risks of the composite (0.82, 0.77–0.88) and mortality (0.71, 0.66–0.78) outcomes than a result in the normoglycemic range.

**Conclusions:**

Previously screened individuals with diabetes had lower risks of cardiovascular events and mortality versus previously unscreened individuals. Better risk assessment tools are needed to support wider and more appropriate uptake of diabetes screening, especially among young adults.

**Supplementary Information:**

The online version contains supplementary material available at 10.1186/s12933-023-01952-y.

## Introduction

Screening for diabetes is performed to proactively identify diabetes before symptoms are clinically apparent, so that early interventions can be enacted to prevent complications [1–3]. Diabetes screening is recommended and widely practiced in many jurisdictions [4]. For example, US guidelines recommend targeting screening to those aged ≥ 35 or with risk factors (e.g., overweight, obesity) [2, 3], while Canadian guidelines recommend targeting screening to those aged ≥ 40 years or with a high risk of diabetes based on a risk calculator [1]. In these targeted populations, screening is likely to reduce the incidence of cardiovascular complications [4,  5],﻿ while screening the entire population does not yield similar benefits [6].

There are risks and benefits to the screening and early detection of diabetes. Potential risks include labeling individuals with a diagnosis associated with higher insurance premiums, and stress stemming from knowledge that one is at high risk of experiencing adverse complications. Benefits include the opportunity to enact interventions to prevent, delay, or manage diabetes by addressing cardiometabolic risk factors [3]. People at high risk of diabetes and their health care providers might be more likely to support screening in appropriate high-risk populations if they knew that screening had benefits, and that lack of screening was associated with risks to health. We conducted an observational study among adults with incident diabetes to examine the association of prior outpatient diabetes screening with the risks of cardiovascular events and mortality in Ontario, Canada. We hypothesized that prior outpatient diabetes screening is associated with lower risks of these outcomes.

## Methods

### Study design and setting

This was a population-based cohort study using health administrative databases in Ontario, the most populous province of Canada. Permanent residents of Ontario receive physician and hospital services through the publicly-funded Ontario Health Insurance Plan (OHIP).

### Study population

We included permanent residents aged ≥ 20 years with incident non-gestational diabetes diagnosed between January 1, 2014 and December 31, 2016. We excluded individuals with any previous hospitalization for myocardial infarction, stroke, coronary revascularization (percutaneous coronary intervention or coronary artery bypass graft surgery).

### Data sources

We used the Ontario Diabetes Database (ODD), which includes Ontario residents with physician-diagnosed diabetes. Non-gestational diabetes was identified by having 3 OHIP physician billing claims for diabetes within 1 year (validated positive predictive value 91.4%), excluding claims occurring within 120 days before or 180 days after a pregnancy-related hospitalization [7]. Cases with a diagnosis date prior to January 1, 2014 were excluded, to ensure that only incident cases were included. Outpatient laboratory testing results were retrieved from the Ontario Laboratories Information System (OLIS) database, a province-wide, centralized repository of results from community, hospital, and public health laboratories [8].

Demographic information was obtained from the Registered Persons Database (RPDB). History of cardiovascular disease, other baseline co-morbidities and primary care utilization (defined as the number of visits to a family doctor in the year prior to diabetes diagnosis) were determined from the Canadian Institutes of Health Information (CIHI) Discharge Abstract Database, the CIHI Same-Day Surgery database, OHIP database, National Ambulatory Care Reporting System and Ontario Mental Health Reporting System (Additional file 1: Table S1). Since we did not have information on individual-level smoking status and income [9], neighbourhood-level smoking rates and income levels from the Canadian Community Health Survey (CCHS) and Canadian census data were used as proxies. All datasets were linked using unique, encoded identifiers and analyzed at ICES.

### Exposure

As physician-diagnosed diabetes was required for entry into the cohort, all individuals in the study would have undergone some form of diabetes testing by default. However, our exposure of interest was outpatient diabetes screening performed *prior* to the diagnosis of diabetes. Diabetes Canada recommends that those targeted for screening should be tested every 3 years in general, or more frequently for individuals with an especially high risk of diabetes [1]. Therefore, we defined prior outpatient screening as having *an outpatient diabetes screening test performed in the 3 years preceding the diagnosis of diabetes* (Additional file 1: Figure S1). We excluded any screening tests occurring within 90 days before the date of diabetes diagnosis (“washout” period), based on our assumption that tests within this period would have contributed directly to the diagnosis of diabetes [10]. For example, an individual who received outpatient diabetes screening for the first time, and was diagnosed with diabetes within the next 90 days, would be classified as having no *prior* outpatient screening. If more than one eligible screening test was performed, then we used the result of the most recent eligible test only.

We included all 3 tests for diabetes screening: glycated hemoglobin (HbA_1c_), fasting plasma glucose (FPG), and 2-h plasma glucose after 75 g oral glucose tolerance test (OGTT) [11]. The most recent *prior* outpatient diabetes screening test result was classified as “normoglycemic range” (HbA_1c_ < 6.0%, FPG < 6.1 mmol/L, OGTT < 7.8 mmol/L), “prediabetes range” (HbA_1c_ 6.0–6.4%, FPG 6.1–6.9 mmol/L, OGTT 7.8–11.0), and “diabetes range” (HbA_1c_ ≥ 6.5%, FPG ≥ 7.0 mmol/L, OGTT ≥ 11.1 mmol/L) [12]. Classification in the “diabetes range” on a prior screening test was not considered as a diagnosis of diabetes, because diagnosing diabetes requires at least 2 positive tests on different days, in the absence of symptomatic hyperglycemia [11]. The primary exposure variable was prior screening versus no prior screening. The secondary exposure variable was prior screening result (diabetes range, prediabetes range, normoglycemic range), limited to those who received prior screening. We excluded tests performed during the last 20 weeks of pregnancy or during hospitalization, as our focus was non-pregnant outpatient diabetes screening.

### Outcomes

The co-primary outcomes were (1) a composite of all-cause mortality and hospitalization for myocardial infarction, stroke, coronary revascularization (percutaneous coronary intervention or coronary artery bypass graft surgery), and (2) all-cause mortality. Secondary outcomes included each component of the co-primary composite outcome aside from mortality, hospitalization for heart failure, and hospitalization for unstable angina. We followed individuals to the earliest occurrence of the outcome, death, departure from the province, and December 31, 2018 (final follow-up date).

### Statistical analysis

We described the baseline characteristics of the study population. Rates of each outcome were calculated as age and sex-standardized rates per 1000 person-years of follow-up, standardized to the 2016 Ontario census population. We used Cox proportional hazards models to determine the adjusted hazard ratios (HR) for each of the exposures with mortality. We adjusted for pre-specified, clinically significant confounding variables including age, sex, neighbourhood income quintile, hypertension, dyslipidemia, neighbourhood smoking, cancer, asthma or COPD, peripheral vascular disease, dementia, liver disease, chronic kidney disease, primary care utilization, and hospitalization or emergency department visits for mood or psychotic disorders. We repeated the analysis for the other outcomes, using cause-specific hazard models to account for the competing risk of death [13]. Because screened individuals with prior normoglycemia had higher than expected mortality rates, we conducted an exploratory post-hoc analysis of their causes of death. All analyses were performed using SAS version 9.4 (SAS Institute, Cary, NC) and 2-sided p-values < 0.05 considered significant. The use of the data in this project is authorized under section 45 of Ontario’s Personal Health Information Protection Act (PHIPA) and does not require review by a Research Ethics Board.

## Results

Baseline characteristics are summarized in Table 1. We identified 178,753 Ontarians with incident diabetes, most (70.2%; *n* = 125,425) of whom received outpatient screening prior to diabetes diagnosis. The median follow-up time was 3.4 years (screened, 3.4 years; unscreened, 3.5 years). Individuals receiving prior screening were more likely to be older (mean age: screened, 58.3 years; unscreened, 53.4 years), female (screened: 49.6%; unscreened: 40.0%), and slightly more likely to be residing in urban areas (screened: 91.5%; unscreened: 90.3%) or high-income neighbourhoods (screened: 15.5%; unscreened: 14.3%; p-values < 0.0001 for all comparisons), compared to individuals without prior screening. Previous comorbidities and primary care visits were also more frequent among those who received prior screening than those without prior screening.

**Table 1:** Baseline characteristics of the study population, stratified by prior outpatient diabetes screening test and screening test results

	Prior outpatient screening result			No prior outpatient screening	p-value
	Normoglycemic range	Prediabetes range	Diabetes range		
	N = 25,087	N = 40,483	N = 59,855	N = 53,328	
Age (years; mean ± standard deviation)	55.7 ± 14.5	59.1 ± 12.6	58.9 ± 12.6	53.4 ± 13.6	< 0.001
Female	13,547 (54.0)	20,096 (49.6)	28,575 (47.7)	21,355 (40.0)	< 0.001
Income quintile					
1 (lowest)	6156 (24.6)	9112 (22.5)	13,984 (23.4)	13,562 (25.5)	< 0.001
2	5360 (21.4)	8884 (22.0)	13,401 (22.4)	11,846 (22.3)	
3	5209 (20.8)	8440 (20.9)	12,516 (20.9)	10,773 (20.3)	
4	4468 (17.8)	7467 (18.5)	10,827 (18.1)	9377 (17.6)	
5 (highest)	3838 (15.3)	6518 (16.1)	9046 (15.1)	7604 (14.3)	
Rural residence	1658 (6.6)	3231 (8.0)	5747 (9.6)	5161 (9.7)	< 0.001
Previous comorbidities					
Hypertension	13,176 (52.5)	24,366 (60.2)	34,899 (58.3)	17,280 (32.4)	< 0.001
Dyslipidemia	6202 (24.7)	9453 (23.4)	9936 (16.6)	3360 (6.3)	< 0.001
Chronic kidney disease	970 (3.9)	1189 (2.9)	1760 (2.9)	754 (1.4)	< 0.001
Asthma or COPD	6237 (24.9)	9992 (24.7)	13,768 (23.0)	8572 (16.1)	< 0.001
Cancer	2098 (8.4)	3491 (8.6)	5021 (8.4)	2333 (4.4)	< 0.001
Liver disease	463 (1.8)	476 (1.2)	748 (1.2)	522 (1.0)	< 0.001
Dementia	505 (2.0)	555 (1.4)	732 (1.2)	415 (0.8)	< 0.001
Recent psychiatric hospitalization^a^	763 (3.0)	689 (1.7)	966 (1.6)	749 (1.4)	< 0.001
Primary care visits (median, IQR)^b^	5 (3–9)	4 (2–7)	4 (2–6)	2 (1–4)	< 0.001

All values are counts (and percentages) unless otherwise indicated

*COPD* chronic obstructive pulmonary disease, *IQR* interquartile range

The p-value is for differences across all 4 groups. Income quintile was missing for ≤ 0.3% of individuals

^a^Hospitalization or emergency visit for mood or psychotic disorder including schizophrenia within 1 year before diabetes diagnosis

^b^Within 1 year before diabetes diagnosis

**Fig. 1:**
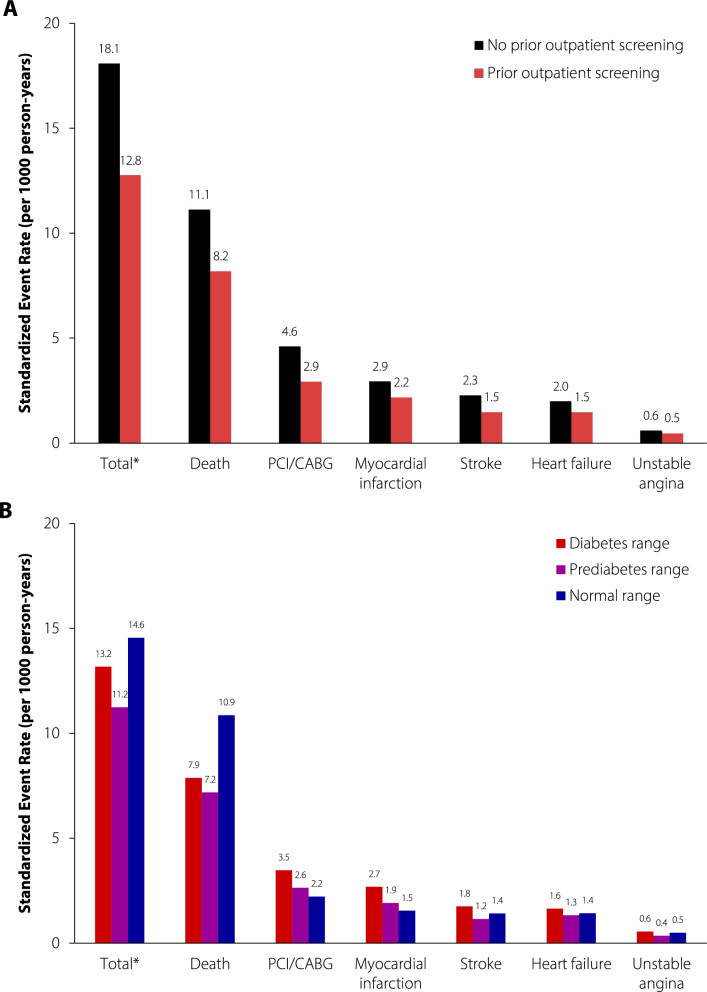
Standardized outcome event rates for individuals with incident diabetes (diagnosed 2014–2016, followed up until 2018), stratified by **A** prior outpatient screening; and **B** prior outpatient screening test result (screened individuals only). All rates are standardized by age and sex to the 2016 Ontario census population. PCI, percutaneous coronary intervention; CABG, coronary artery bypass graft. *primary composite outcome including death, PCI, CABG, myocardial infarction, and stroke

**Fig. 2:**
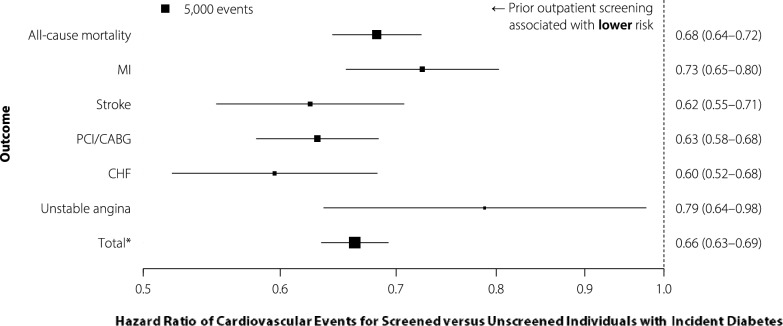
Adjusted hazard ratios (95% confidence intervals) of cardiovascular events for individuals with incident diabetes (diagnosed 2014–2016, followed up until 2018) with a prior outpatient screening test versus those without a prior outpatient screening test. Hazard ratios are adjusted for age, sex, neighbourhood income quintile, hypertension, dyslipidemia, neighbourhood smoking, cancer, asthma or chronic obstructive pulmonary disease, peripheral vascular disease, dementia, liver disease, chronic kidney disease, and number of family physician visits in the year prior to diabetes diagnosis and history of hospitalization or emergency department visit for a mood/psychotic disorder in prior 5 years. The area of each box is proportional to the number of events recorded during the study

Individuals receiving prior screening had relatively lower standardized event rates than those without prior screening across all outcomes (co-primary composite outcome: 12.8 versus 18.1, co-primary mortality outcome 8.2 versus 11.1 per 1000 patient-years; see Fig. 1A for secondary outcomes). In the survival analyses with multivariable adjustment, prior screening was associated with 34% and 32% lower hazards of the co-primary composite outcome (HR 0.66, 0.63–0.69) and the co-primary mortality outcome (HR 0.68, 0.64–0.72; Fig. 2). Similar patterns were observed for the secondary outcomes.

**Fig. 3:**
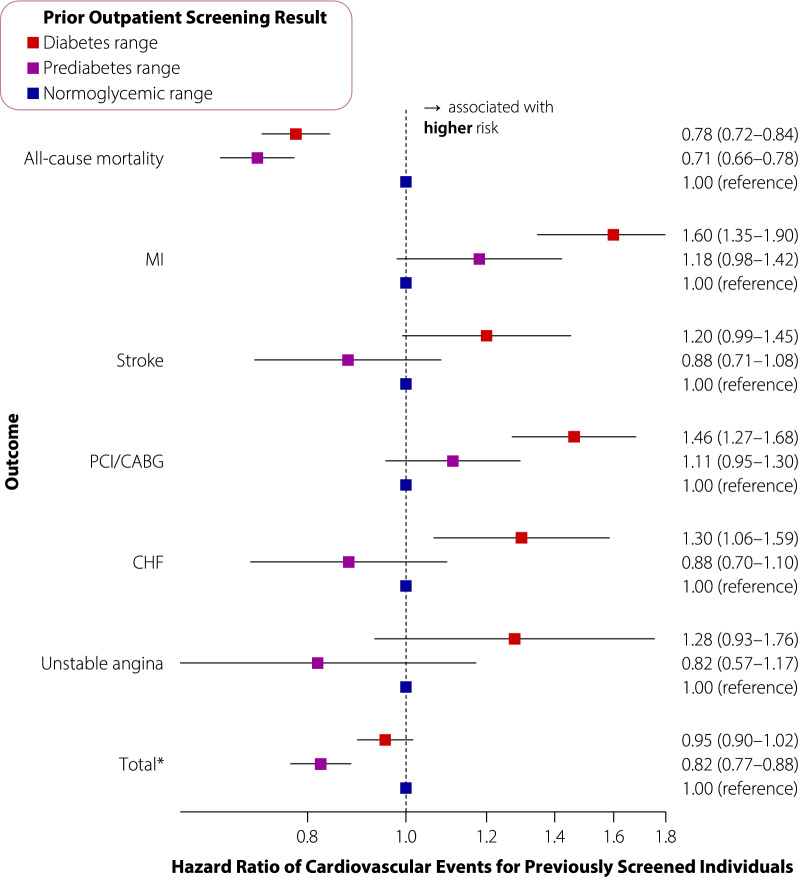
Adjusted hazard ratios (95% confidence intervals) of cardiovascular events for individuals with incident diabetes (diagnosed 2014–2016, followed up until 2018) with a prior outpatient screening test versus, stratified by screening test result. Hazard ratios are adjusted for age, sex, neighbourhood income quintile, hypertension, dyslipidemia, neighbourhood smoking, cancer, asthma or chronic obstructive pulmonary disease, peripheral vascular disease, dementia, liver disease, chronic kidney disease, and number of family physician visits in the year prior to diabetes diagnosis and history of hospitalization or emergency department visit for a mood/psychotic disorder in prior 5 years

The following results pertain to individuals receiving prior screening, stratified by test result. Individuals in the normoglycemic range had the standardized highest rates of the co-primary outcomes (composite: 14.6, mortality: 10.9 per 1000 patient-years; Fig. 1B), followed by those in the diabetes (composite: 13.2, mortality: 7.9 per 1000 patient-years) and prediabetes (composite: 11.2, mortality: 7.2 per 1000 patient-years) ranges. For the secondary outcomes, those in the normoglycemic range had lower event rates than those in the diabetes range, and lower or comparable event rates than those in the prediabetes range (e.g., myocardial infarction: normoglycemic range 1.5, prediabetes range 1.9, diabetes range 2.7, no screening 2.9 per 1000 patient-years). After multivariable adjustment, the prediabetes range was associated with lower risks of the co-primary outcomes versus the normoglycemic range (HR, composite: 0.82, 0.77–0.88; mortality: 0.71, 0.66–0.78; Fig. 3), while the diabetes range was associated with lower mortality (HR 0.78, 0.72–0.84) and a similar risk of the primary composite outcome (HR 0.95, 0.90–1.02) versus the normoglycemic range. The diabetes range was associated with generally higher risks of the secondary outcomes compared to the normoglycemic range, while the prediabetes range was associated with similar risks of the secondary outcomes as the normoglycemic range.

To better understand the higher death rate in individuals with prior screening and normoglycemia, an exploratory analysis of the cause of death was undertaken. The top 4 causes of death were related to cancer (accounting for 19.0% of all deaths; Additional file 1: Table S2).

## Discussion

This multiethnic population-based study in Ontario, Canada—a setting where diabetes screening is recommended and commonly practiced—suggested that prior diabetes screening was associated with lower risks of cardiovascular events and mortality than no prior screening. This pattern was consistent across a variety of cardiovascular outcomes, even after accounting for differences in primary care utilization, previous comorbidities, age, and other sociodemographic factors. Furthermore, we found that prior screening results in the normal or prediabetes ranges were generally associated with lower risks of cardiovascular events than the diabetes range, while the benefit associated with prior normoglycemia was unexpectedly attenuated for all-cause mortality. Although the factors explaining these patterns are unclear, our real-world findings lend support to the practice of diabetes screening, and complement the findings of randomized control trials in single-ethnic populations. In particular, the younger age distribution of previously unscreened individuals suggests that further efforts are required to promote the early detection of young-onset type 2 diabetes (defined as age at diagnosis < 40 years). Discussion of risk assessment tools to support wider uptake of diabetes screening among higher-risk young adults is needed.

Our findings illustrate how variations in screening approaches across trial and real-world settings might affect the age at diagnosis differently. In the Ely randomized controlled trial, diabetes was diagnosed an average of 3.3 years earlier in screened versus unscreened individuals [14]. Similarly, people with undiagnosed diabetes in the UK biobank became clinically diagnosed after a median of 2.2 years [15]. In an observational study in Västerbotten, Sweden, all residents were invited for screening every 10 years from age 30 to 60, and the 1024 individuals with screen-detected diabetes were 4.6 years younger than the 8642 individuals with diabetes detected outside the program [16]. These findings demonstrate how routine screening can diagnose diabetes years before symptoms appear. However, in settings where screening is preferentially targeted to people with cardiovascular risk factors strongly associated with older age (e.g., hypertension), those with screen-detected diabetes are older than those with symptomatically-detected diabetes. For example, a small multicentre Dutch observational primary care study (“Diabscreen”) compared those screened for diabetes based on the presence of diabetes risk factors to those diagnosed clinically after developing symptoms. The 359 individuals with diabetes detected by screening were 2.8 years older and had more baseline cardiovascular comorbidities than the 206 individuals with diabetes detected after developing symptoms [17]. Our much larger study extends these findings by confirming that previously screened individuals were 2–6 years older and had more cardiovascular risk factors than previously unscreened individuals with incident diabetes. This pattern is consistent with the rationale used by the health care providers of these people to initiate screening. This result is also consistent with the Canadian practice recommendation to screen in those aged ≥ 40 years or in high-risk populations [1], and our prior findings of insufficient screening in people aged < 50 years [18]. Our data show that better research, tools, and strategies are needed to ensure that younger people at high risk of diabetes are also appropriately screened and not overlooked—especially as young-onset type 2 diabetes incidence continues to rise worldwide [19].

Nevertheless, our results support diabetes screening in targeted populations. While previous European diabetes screening trials reported negative findings [20–22], the low historical diabetes prevalence rates (3–6%) [21, 23] in these European populations likely limited power. In the Anglo-Danish-Dutch Study of Intensive Treatment In People with Screen Detected Diabetes in Primary Care (ADDITION)–Denmark non-randomized controlled trial, moderate- to high-risk respondents were identified by a questionnaire and invited for screening [24]. Those diagnosed with diabetes in the screening group had a 16–21% lower risk of cardiovascular disease and mortality than the 125,083 people with diabetes in the comparison group [24]. Similarly, among people with diabetes in the Västerbotten study, previously screened individuals had a 35–48% reduction in mortality and cardiovascular disease versus those without prior screening [16]. We found that previously screened individuals with diabetes had 30–40% lower risk of cardiovascular events and mortality versus previously unscreened individuals with diabetes. It is possible that screening prompted healthy behaviour changes, but several other factors might have impacted the results. Those attending screening might have healthier behaviours than the general population (“healthy user bias”) [16], but we observed that previously unscreened people had the least comorbidities, and the associations persisted after accounting for primary care utilization and socioeconomic status. “Lead-time bias” is when the benefits of screening are overestimated because screened individuals are identified at an earlier stage of disease. The influence of this bias is unclear, as we classified people based on prior screening, and previously screened people were older than previously unscreened people. However, younger age (< 40 years) at diagnosis is associated with more rapid progression and increased renal and other complications than older age [25, 26]. “Length–time bias” occurs when slowly-progressing cases (e.g., older adults) have a longer asymptomatic period and thus a higher likelihood of being detected by screening than rapidly progressive cases (e.g., younger adults), which may only be detected after symptoms occur. Future studies can explore how targeted approaches to screening, based heavily on age, might contribute to delayed identification and poor outcomes of people with young-onset type 2 diabetes.

Among previously screened individuals, we revealed unexpected differences across the mortality and cardiovascular outcomes. In particular, those with previous normoglycemia had a high standardized mortality rate comparable to previously unscreened individuals. It is possible for previously normoglycemic individuals to develop rapidly progressive diabetes in the context of underlying terminal comorbidities or treatments (e.g., advanced liver disease, pancreatic cancer, cancer treatments including glucocorticoids) [27, 28]. Accordingly, we found that the risk of mortality in previously normoglycemic individuals was relatively attenuated after accounting for cancer, liver disease, and other comorbidities. Interestingly, post-hoc analyses revealed that pancreatic cancer was the second-most common cause of death in this group (Additional file 1: Table S2). By contrast, those previously found to be in the normal, pre-diabetes, and diabetes ranges had stepwise increases in risk of cardiovascular outcomes after diabetes diagnosis, matching the known gradient of risk associated with these states [29]. Although this pattern is inconsistent with a more rapid rate of progression in previously normoglycemic individuals, it is possible that healthy behaviours may have benefit for cardiovascular, but not non-cardiovascular, outcomes. Further research will help to understand how prior screening affects health-related behaviours, the intensity of preventive management of cardiovascular risk factors, and the subsequent rate of progression to diabetes in the presence of various types of comorbidities.

The strengths of this study include its real-world population-based design, multiethnic setting with wide uptake of diabetes screening, large sample size allowing for well-powered examination of a variety of outcomes, and competing risk analysis. Limitations include the aforementioned healthy user, lead-time, and length–time biases. Potential misclassification due to factors such as missing laboratory data is less likely to impact as around 95% of laboratory results were captured, and such misclassification would have biased our findings in the conservative direction [30]. We lacked information on medications and diabetes type, but > 95% of individuals likely had type 2 diabetes [31].

In summary, our large population-based study adds important observational evidence to support the hypothesis that real-world, multiethnic populations benefit from earlier identification of diabetes by screening, and its association with reduced complications. The 30% of individuals diagnosed with diabetes without screening had no opportunity to receive risk factor modification, and better approaches are needed to improve timely identification of type 2 diabetes**,** particularly in younger people.

### Supplementary Information

**Additional file 1: Figure S1.** Timeframe definitions for the study. **Table S1**. Data sources, diagnostic codes, and other criteria for identification of comorbidities, outcomes, and glucose testing. **Table S2** Exploratory post-hoc analysis of the 10 most common causes of death.

## Data Availability

The data set from this study is held securely in coded form at ICES. While data sharing agreements prohibit ICES from making the data set publicly available, access may be granted to those who meet pre-specified criteria for confidential access, available at http://www.ices.on.ca/DAS.
