# Supplementary material for: Association of prior outpatient diabetes screening with cardiovascular events and mortality among people with incident diabetes: a population-based cohort study
Source: Cardiovasc Diabetol. 2023 Aug 28;22:227. doi: 10.1186/s12933-023-01952-y (PMC10463666; doi:10.1186/s12933-023-01952-y)
Supplement: Supplementary file 1 — Additional file 1: Figure S1. Timeframe definitions for the study. Table S1. Data sources, diagnostic codes, and other criteria for identification of comorbidities, outcomes, and glucose testing. Table S2 Exploratory post-hoc analysis of the 10 most common causes of death. [file 12933_2023_1952_MOESM1_ESM.pdf]

Additional file

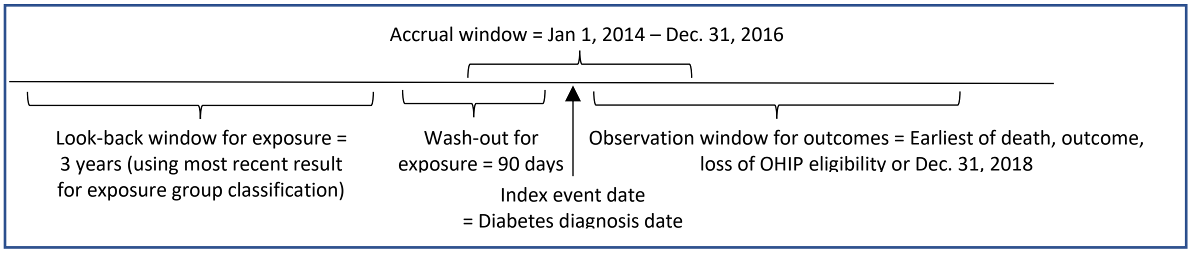

Figure S1 Timeframe definitions for the study

Table S1. Data sources, diagnostic codes, and other criteria for identification of comorbidities, outcomes, and glucose testing

**Comorbidity / Outcome**	**Data source(s)**	**Codes**
Asthma	CIHI DAD	ICD-9: 493<br>ICD-10-CA: J45, J46
	OHIP	ICD-9: 493
Cancer	OCR	Any record in registry, except non-melanoma skin cancers
Chronic kidney disease (prior 5 years)	CIHI DAD/SDS	ICD-10-CA: E102, E112, E132, E142, I12, I13, N08, N18, N19
	OHIP	ICD-9: 403, 585
Chronic obstructive pulmonary disease	CIHI DAD	ICD-9: 491, 492, 496<br>ICD-10-CA: J41, J42, J43, J44
	OHIP	Diagnosis codes: 491, 492, 496
Coronary revascularization (percutaneous coronary intervention or coronary artery bypass graft surgery)*	CIHI DAD	CCP: 4802, 4803, 481<br>CCI: 1IJ50, 1IJ54, 1IJ57GQ, 1IJ76
Death	RPDB	Not applicable
Dementia	CIHI DAD/SDS	ICD-9: 46.1, 290.0-290.4, 294, 331.0, 331.1, 331.5<br>ICD-10-CA: F00, F01, F02, F03, G30
	OHIP	ICD-9: 290, 331
	ODB	Prescription for any cholinesterase inhibitor
Dyslipidemia	OHIP	ICD-9: 272 (2 within 2 years)

Heart failure*	CIHI DAD	ICD-9: 428<br>ICD-10-CA: I50
Hypertension	CIHI DAD/SDS	ICD-9: 401, 402, 403, 404, 405<br>ICD-10-CA: I10, I11, I12, I13, I15
	OHIP	ICD-9: 401, 402, 403, 404, 405
Liver disease	CIHI DAD	Mild:<br>ICD-9: 0702, 0703, 0704, 0705, 0706, 0709, 570, 571, 5733, 5734, 5738, 5739, V427<br>ICD-10-CA: B18, K700-K703, K709, K713-K715, K717, K760, K762-K764, K768, K769, K73, K74, Z944
		Moderate or severe:<br>ICD-9: 4560-4562, 5722-5728<br>ICD-10-CA: I850, I859, I864, I982, K704, K711, K721, K729, K765, K766, K767
Mood, schizophrenia spectrum or other psychotic disorder in the prior 5 years[a]	OMHRS	DSM 5: 291.x, 292.x, 303.x, 304.x, 305.x, 312.31 with provisional DSM 5=16; or DSM 5: 293.83, 296.x, 300.4x, 301.13, 311.x, 625.4. with provisional DSM 5=3, 4
	CIHI DAD, NACRS	ICD-10-CA: F06.0-3, F20, F22-F29, F30.x-F34.x, F38.x, F39.x, F53.0, F53.1
Myocardial infarction*[b]	CIHI DAD	ICD-9: 410<br>ICD-10-CA: I21, I22
Number of visits to a family doctor in the year prior to diabetes diagnosis	OHIP	Non-lab visits with specialist code = '00' (Family practice and general practice) and location code = 'O' (office), 'L' (long-term care) or 'H' (home)
Peripheral vascular disease[c]	CIHI DAD	ICD-9: 0930, 4373, 440, 441, 4431-4439, 4471, 5571, 5579, V434<br>ICD-10-CA: I70, I71, I731, I738, I739, I771, I790, I792, K551, K558, K559, Z958, Z959
Stroke*[d]	CIHI DAD	ICD-9: 430, 431, 434, 436, 362.3<br>ICD-10-CA: I60, I61, I63 (excluding I63.6), I64, H34.1
Unstable angina[b]	CIHI DAD	ICD-10-CA: I20
**Glucose testing, included any of the following:**		
1. Glycosylated hemoglobin<br>2. Quantitative plasma/serum glucose<br>3. Oral glucose tolerance test	OLIS	LOINC 17855-8, 17856-6, 41995-2, 4548-4, 71875-9<br>LOINC 14771-0<br>Record with each of the following on the same test order:

		1. Fasting glucose: LOINC 1552-9, 14749-6, 14771-0, 14996-3, 15074-8, 39480-9, or 47622-6<br>2. 2-hr glucose: LOINC 14759-5 or 14995-5<br>3. At least one of the following:<br>a. Fasting glucose value with LOINC 1552-9 or 1496-3<br>b. 2-hr glucose value with LOINC 14995-5<br>c. 75 g glucose dose: LOINC 4269-7 record with a value of 75

Abbreviations: CCI, Canadian Classification of Health Interventions; CCP, Canadian Classification of Diagnostic, Therapeutic, and Surgical Procedures; CIHI, Canadian Institute for Health Information; DAD, Discharge Abstract Database; DSM 5, Diagnostic and Statistical Manual of Mental Disorders; ICD, International Classification of Diseases; LOINC, Logical Observation Identifiers Names and Codes; NACRS, National Ambulatory Care Reporting System; OCR, Ontario Cancer Registry; OHIP, Ontario Health Insurance Plan; ODB, Ontario Drug Benefit database; OLIS, Ontario Laboratories Information System; OHMRS, Ontario Mental Health Reporting System; RPDB, Registered Persons Database; SDS, Same-Day Surgery Database

*Only ICD-10-CA and CCI codes were used for outcome ascertainment

[a]MHASEF Research Team. Mental Health and Addictions System Performance in Ontario: A Baseline Scorecard. Toronto, ON: Institute for Clinical Evaluative Sciences; 2018. Available from: https://www.ices.on.ca/Publications/Atlases-and-Reports/2018/MHASEF.

[b]Austin PC, Daly PA, Tu J V. A multicenter study of the coding accuracy of hospital discharge administrative data for patients admitted to cardiac care units in Ontario. American heart journal. 2002 Aug;144(2):290–6.

[c]Deyo RA, Cherkin DC, Ciol MA. Adapting a clinical comorbidity index for use with ICD-9-CM administrative databases. Journal of clinical epidemiology. 1992 Jun;45(6):613–9.

[d]Kokotailo RA, Hill MD. Coding of stroke and stroke risk factors using international classification of diseases, revisions 9 and 10. Stroke. 2005 Aug;36(8):1776–81.

Table S2 Exploratory post-hoc analysis of the 10 most common causes of death (International Classification of Diseases version 10 codes) among people with normoglycemia on prior screening (total *n*=737), based on linkage to cause of death data from the Office of the Registrar General

Cause of Death	*n*	%
C34: Malignant neoplasm of bronchus and lung	38	5.2
C25: Malignant neoplasm of pancreas	37	5.0
C79: Secondary malignant neoplasm of other sites	33	4.5
C80: Malignant neoplasm without specification of site	32	4.3
F03: Unspecified dementia	31	4.2
I25: Chronic ischemic heart disease	29	3.9
J18: Pneumonia, organism unspecified	23	3.1
J44: Other chronic obstructive pulmonary disease	16	2.2
G30: Alzheimer’s disease	13	1.8
I21: Acute myocardial infarction	12	1.6
